# Pharmacokinetics and efficacy of a ketorolac-loaded ocular coil in New Zealand white rabbits

**DOI:** 10.1080/10717544.2021.1883157

**Published:** 2021-02-17

**Authors:** Christian J. F. Bertens, Marlies Gijs, Aylvin A. J. Dias, Frank J. H. M. van den Biggelaar, Arkasubhra Ghosh, Swaminathan Sethu, Rudy M. M. A. Nuijts

**Affiliations:** a Chemelot Institute for Science and Technology (InSciTe), Maastricht, The Netherlands; b University Eye Clinic Maastricht, Maastricht University Medical Center+ (MUMC+), Maastricht, The Netherlands; c Eyegle bv, Maastricht, The Netherlands; d GROW Research Laboratory, Narayana Nethralaya Foundation, Bangalore, India

**Keywords:** Ketorolac tromethamine, ocular coil, pharmacokinetics, ocular drug delivery device, sustained drug delivery, anti-inflammation

## Abstract

Eye drops are considered standard practice for the delivery of ocular drugs. However, low patient compliance and low drug levels compromise its effectiveness. Our group developed a ketorolac-loaded ocular coil for sustained drug delivery up to 28 days. The aim of this study was to gain insight into the pharmacokinetics and efficacy of the ocular coil. The pharmacokinetics of the ketorolac-loaded ocular coil versus eye drops were tested in New Zealand White rabbits by repetitive sampling for 28 days. Efficacy of the ocular coil was also tested in New Zealand White rabbits. Ocular inflammation was induced where after the ocular coil was inserted, or eye drops, or no treatment was provided. The total protein concentration and cytokine levels were measured in tears, aqueous humor, and plasma at 4 h, 8 h, 24 h, 4 d, 7 d, 14 d, 21 d, and 28 d. Four h after inserting the ocular coil in the eye, ketorolac levels in aqueous humor and plasma were higher in the ocular coil group than in the eye drop group. Ketorolac released from the ocular coil could be detected up to 28 d in tears, up to 4 d in aqueous humor and up to 24 h in plasma. After inducing inflammation, both the ocular coil and eye drops were able to suppress prostaglandin E_2_, TNFα and IL-6 levels in aqueous humor and plasma as compared to the group that received no treatment. To conclude, the ocular coil facilitated a sustained release of the drug and showed similar therapeutic benefit in suppressing post-operative inflammation as eye drops.

## 1. Introduction

Topical administration of eye drops is the most commonly prescribed treatment strategy in the prevention and treatment of ocular disorders (Urtti, 2006). Despite the achieved therapeutic concentrations in anterior segment tissues, eye drops have significant disadvantages. A short duration of action, high peak drug concentrations, and considerable systemic absorption of the drug are several important shortcomings of eye drops (Bertens et al., 2018). To maintain minimal effective concentrations (MEC), drugs need to be dosed frequently. However, it is known that patient compliance (the degree to which a patient correctly follows medical advice) of eye drops is low (Olthoff et al., 2009; Eaton et al. 2015; Mohindroo et al., 2015; Farkouh et al., 2016). Frequently reported reasons for noncompliance include forgetfulness (26.7% of patients treated with eye drops), limited access to eye drops (20%), and insufficient ability to properly self-instil the eye drops (16.2%) (Olthoff et al., 2009). As a result of low compliance, the effectivity of the prescribed therapy is compromised.

To improve drug delivery and bypass patient compliance issues, injections (subconjunctival, subtenon, intracameral, intravitreal) into the target site can be used. However, injections only deliver a single (high) dose of drugs at a single time point to the affected eye. Furthermore, injections are invasive and can be accompanied with complications or side effects. Therefore, new methods for ocular drug delivery are essential within the ophthalmic field.

In addition to *in vitro* drug release studies, *in vivo* studies are needed to determine the pharmacokinetics, pharmacodynamics, and the MEC of the delivered drugs in a complete system. Based on these values, application regimes can be optimized and safety of the drugs (and the additives) can be assured.

To improve ocular drug delivery, our group developed an ocular coil that can be placed in the lower conjunctival fornix (Pijls et al., 2004, 2005, 2006, 2007; Bertens et al., 2020). The ocular coil consists of a coiled and coated wire, closed on both extremities with a dome-shaped cap. The ocular coil is filled with a non-steroidal anti-inflammatory drug (NSAID), ketorolac tromethamine, containing microspheres in its inner lumen to serve as a slow-release drug delivery device. In our previous study, we show *in vitro* release of ketorolac for 28 days from the ocular coil (Bertens et al., 2020).

In this preclinical study, we investigate the pharmacokinetics of a ketorolac-loaded ocular coil, and tested its efficacy of suppressing inflammation after surgical trauma in New Zealand White rabbits. Surgical trauma was mimicked by a paracentesis of the anterior chamber.

## 2. Materials and methods

### 2.1. The ocular coil

The technical details and in vitro release kinetics have been previously described (Bertens et al., 2020). Briefly, ocular coils (16 mm long, wire thickness of 0.084 mm with an outer diameter of 0.90 mm) were ordered from EPflex (Dettingen an der Erms, DE). The ocular coils were manually filled with 3 mg ketorolac entrapped poly-methyl methacrylate (PMMA, *M*_n_ ≈ 43 kg/mol) microspheres (26.5 wt% drug loading) 150 µm ± 10 µm in diameter. Hereafter, the ocular coil was closed on both extremities with a dome-shaped UV-curable acrylate urethane cap to soften its extremities while maintaining the drug-eluting matrix inside. The *in vitro* release kinetic study showed that a total of 69.9 ± 5.6% (0.795 ± 0.063 mg ketorolac) of the loaded ketorolac was released in 28 days. In the first 3 days, a high (burst) release of approximately 50% of ketorolac was observed followed by a more gradual release up to 28 days.

### 2.2. Ethics

All animal procedures were conducted according to the Association for Research in Vision and Ophthalmology (ARVO). Statement for the Use of Animals in Ophthalmic and Visual Research and the Guidelines of the Central Laboratory Animal Facility of Maastricht University. All protocols were approved by the Central Authority for Scientific Procedures on Animals (CCD, Den Haag, the Netherlands) and were in accordance with the European Guidelines (2010/63/EU).

### 2.3. Animals

Adult New Zealand White (NZW) rabbits (2.0–2.5 kg, males and females, strain: Hsdlf:NSW) were ordered from Envigo (Horst, NL) and housed in group housing, males and females separated with a maximum of five rabbits per cage (size:4 m^2^). The rabbits had *ad libitum* access to water (regular tap water) and dried animal chow (200 g per animal). After arrival, the animals received one week of acclimatization to the new environment.

**Figure 1.**
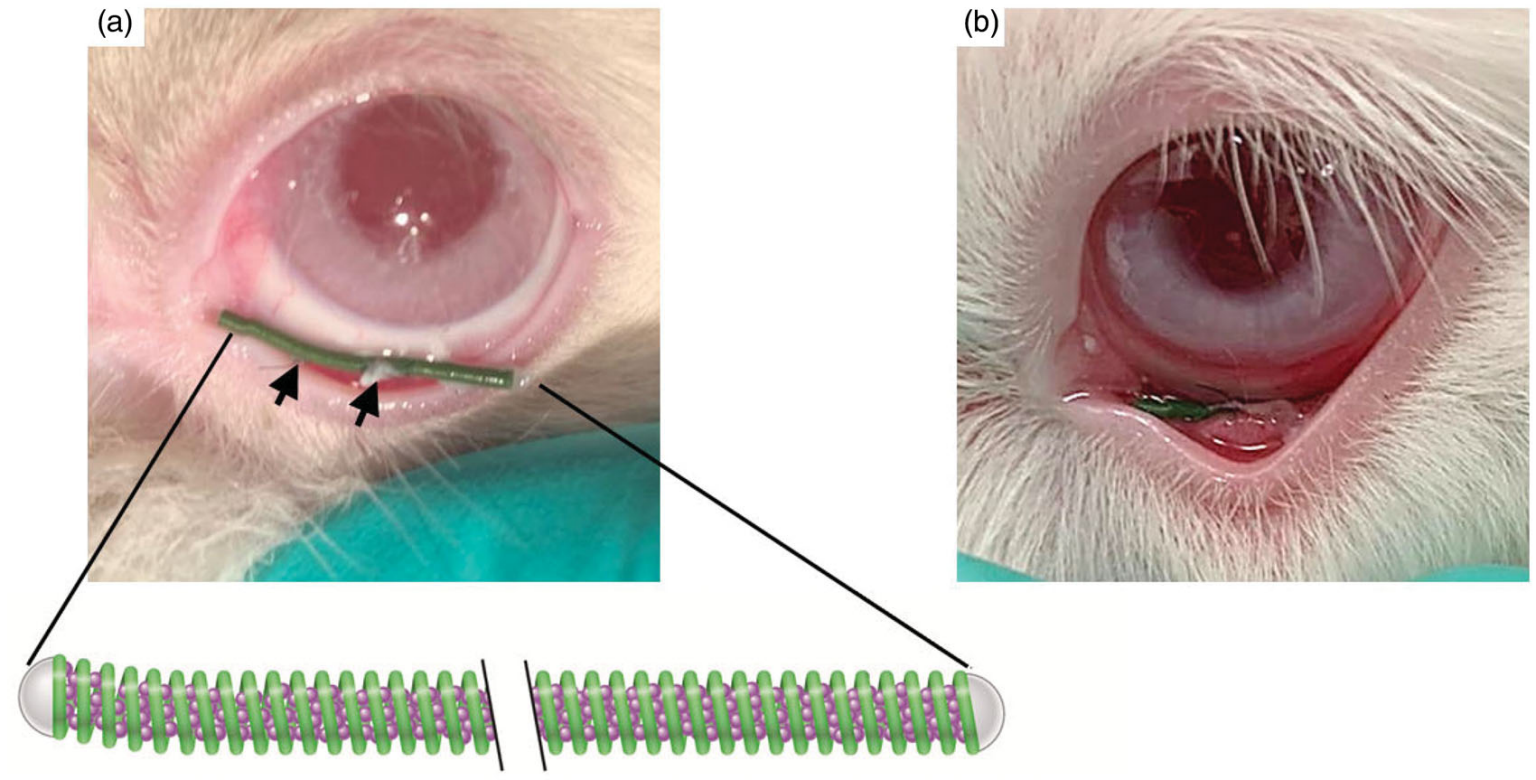
(a) Location of the ocular coil in the conjunctival fornix during the stitching procedure. The arrows indicate two of the three stitches. The magnification below shows a representation of the ocular coil and its microsphere filling. (b) Location of the ocular coil in the conjunctival inferior fornix during normal wear.

During the first experimental procedure (stitching), rabbits were intramuscularly (IM) sedated using ketamine (50 mg/kg) (Alfasan Nederland BV, Woerden, NL) and midazolam (0.5 mg/kg) (Actavis, Dublin, IR). Additionally, they received topical anesthesia using MINIMS® Oxybuprocaine hydrochloride (Bausch & Lomb Pharma, Brussels, BE). Because of the nictitating membrane in rabbits, the ocular coil was stitched into the conjunctival fornix using nylon 8–0 12” stitches (Alcon Inc., Genève, CH). The first stitch was placed centrally, followed by one stitch nasally and one stitch temporally from the first stitch (Figure 1). The other groups also received three stitches without an ocular coil.

During the follow-up moments, rabbits were sedated using medetomidine (1 mg/kg) (A.S.T. Farma BV, Oudewater, NL). After the final sampling at day 28, the rabbits were euthanized using 20% sodium pentobarbital (200 mg/kg) (Euthasol®, Alfasan Nederland BV, Woerden, NL) intravenously (IV) injected.

### 2.4. Treatment groups

Rabbits from the ocular coil group received one ketorolac-loaded ocular coil in the conjunctival fornix of their right eye. The eye drop group received 50 µL ketorolac ophthalmic solution (Acular™, 0.5% ophthalmic ketorolac solution (5 mg/mL), Allergan, Dublin, IR) in the conjunctival fornix of their right eye immediately, 4 h, and 10 h after the stitching procedure. During the following 27 days, these rabbits received eye drops three times daily. Rabbits from the control group did not receive any treatment.

Samples of aqueous humor, tears, and blood from the rabbits of the pharmacokinetic study were drawn at 4 and 24 h, and at days 4, 7, and 28 after stitching. Samples of aqueous humor, tears and blood of the rabbits from the efficacy study were drawn at 4, 8, and 24 h, and at days 4, 7, 14, 21, and 28 after trauma induction.

### 2.5. Induction of inflammation

Inflammation was induced by removing a large volume (approximately 150–175 µL) aqueous humor via a corneal paracentesis as previously described by Unger et al. using a 1 mL insulin syringe and a 29 G needle (Becton Dickinson BV, Vianen, NL) (Unger et al., 1975). Caution was taken not to touch the lens or iris during the procedure. The collected aqueous humor was stored in a 1.5 mL Eppendorf vial at −80 °C.

### 2.6. Sample collection

Tears were sampled from the right eye of the rabbits using Schirmers’ TEARstrips (Contacare Ophthalmics & Diagnostics, Gujarat, IN). The Schirmer’s strips were placed in the inferior conjunctival fornix for 5 min or until complete absorption. Hereafter, the Schirmer’s strips were placed in a 1.5 mL Eppendorf vial and frozen at −80 °C until further treatment. Hereafter, about 3 mL blood was collected via the marginal ear vein into a 5 mL EDTA vacuette tubes (VWR, Amsterdam, NL). After sampling, the vacuette tubes were centrifuged 1500*g* for 10 min at 4 °C. Plasma was gently pipetted off and frozen at −80 °C. This was followed by anterior chamber paracentesis. The paracentesis was performed with a 1 mL insulin syringe (29*g*) (Becton Dickinson BV, Vianen, NL). During the sampling, a small volume (approximately 50 µL) aqueous humor was drawn and frozen at −80 °C until further use. Caution was taken to avoid touching the lens or iris.

### 2.7. Protein and ketorolac extraction from tears

Tears were extracted from the Schirmer’s TEAR strips as described earlier by Sharma et al. (2019). Briefly, the strips were cut into 1 mm pieces and soaked in 200 µL PBS (pH 7.4) for protein extraction, or in 200 µL methanol (99.9% pure, HPLC grade) (VWR, Amsterdam, NL) for ketorolac extraction. This was agitated at 900 rpm (Thermomixer, Eppendorf, Hamburg, DE) at 4 °C for 90 min. Paper was filtered off and collected tear fluid was used for further experiments. The measured concentrations (ketorolac, proteins, and cytokines) were corrected for the tear migration length and dilution to obtain the corrected concentration per milliliter.

### 2.8. Ketorolac detection

Aqueous humor and plasma were diluted four times with methanol (99.9% pure, HPLC grade) (VWR, Amsterdam, NL) and centrifuged for 5 min at 15,000 G at 4 °C to remove proteins. Methanol extracted tears were used without further dilution. The samples were analyzed by HPLC (Agilent 1260 infinity series with EZchrom software, Agilent inc., Santa Clara, CA). Analysis was done according to the US Pharmacopeia (2018), using an elution time of 20 minutes and injection volume of 10 µL, peak UV-detection at 313 nm on a symmetry C18 column (300 Å, 5 µm, 4.6 mm x 250 mm; #WAT106151, Waters corp., Milford, MA) with a symmetry C8 VanGuard pre-column (100 Å, 5 µm, 3.9 mm × 5 mm, 3/pkg, #186007739, Waters corp., Milford, MA). Ketorolac had a retention time of 10.5 min, a limit of detection (LOD) of 4 ng/mL, and a limit of quantification (LOQ) of 10 ng/mL (Bertens et al., 2019). All samples were analyzed in duplicate.

### 2.9. Total protein and inflammatory factor determination

The total protein concentration was determined using BCA protein assay (ThermoFisher Scientific, Waltham, MA). Enzyme-linked immunosorbent assays (ELISAs) were used for the determination of prostaglandin E_2_ (PGE_2_), tumor necrosis factor α (TNFα), interleukin (IL)-6, and IL-1β concentration in aqueous humor, plasma, and tears. PGE_2_ was determined using the Biotrak™ EIA kit (#GERPN222, Merck KGaA, Darmstadt, DE). Samples were diluted 1:4 using assay buffer and a total of 50 µL diluted sample was loaded per well. TNFα, IL-6, and IL-1β were determined using R&D systems DuoSet (#DY5670, #DY7984, #DY7464, R&D Systems, Inc., McKinley Place, MN). Samples were also diluted 1:4 using reagent diluent and 50 µL diluted sample was loaded per well. The assays were performed in singlicate due to limited sample volume.

### 2.10. Statistical analysis

Differences in drug concentration between treatment groups were tested using unpaired student t-test. Samples below the detection limit of ketorolac (4 ng/mL) were set to a value of 4 ng/mL.

For the protein and cytokine assays, outliers were excluded using the robust regression and outlier removal (ROUT) method with a Q of 1% (Motulsky & Brown, 2006). Differences in the total protein concentrations between treatment groups were tested for each time point using Tukey’s single-step multiple comparison procedure. Furthermore, Dunnett’s test was performed for pairwise comparisons of multiple time point to baseline.

All tests were performed using GraphPad Prism version 8 (GraphPad Software Inc. San Diego, CA).

## 3. Results

### 3.1. Pharmacokinetics of the ocular coil versus eye drops

**Figure 2.**
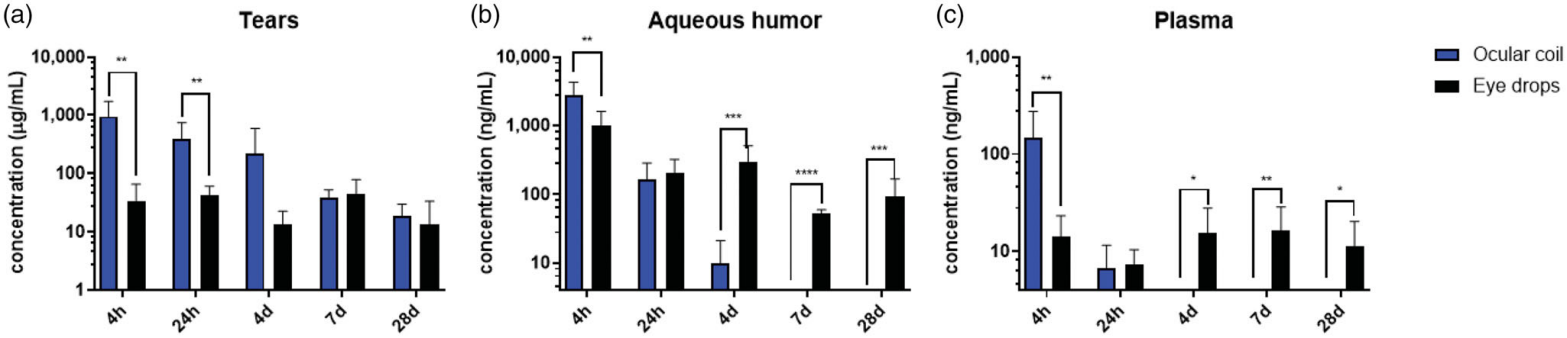
Pharmacokinetics of the ocular coil. Concentration ketorolac detected in (a) tears, (b) aqueous humor, and (c) plasma. *N* = 9 rabbits per group, data are plotted as mean ± SD. * *p* < .05, ** *p* < .01, *** *p* < .001, and **** *p* < .0001.

The pharmacokinetics of the ketorolac-loaded ocular coil was evaluated by measuring the ketorolac concentration in tears, aqueous humor, and plasma at multiple time points (Figure 2). The ketorolac concentration released by the ocular coil at 4 h in tears, aqueous humor, as well as plasma was significantly higher compared to the concentration delivered by the eye drops.

At 4 hours, the ketorolac tear concentration in the ocular coil group was 28 times higher than in the eye drop group (950 ± 782 µg/mL compared to 34 ± 32 µg/mL, respectively, *p* = .003). At 24 h, the tear ketorolac concentration in the ocular coil group was about nine times higher than in the eye drop group (397 ± 348 µg/mL compared to 44 ± 17 µg/mL respectively, *p* = .008). During the first 4 days, the ketorolac concentration in tears (Figure 2(a)) in the ocular coil group was higher than in the eye drop group. At days 7 and 28, the concentration in tears in the ocular coil group was equal to that of eye drops (39 ± 14 µg/mL at day 7 and 19 ± 12 µg/mL at day 28 compared to 44 ± 35 µg/mL at day 7 and 13 ± 20 µg/mL at day 28 for the ocular coil group and the eye drop group, respectively).

In aqueous humor (Figure 2(b)), the ketorolac concentration at 4 hours was significantly higher (*p* = .004) for the ocular coil group compared to the eye drop group (2780 ± 1485 ng/mL and 983 ± 629 ng/mL, respectively). At 24 h, the ketorolac concentration of the ocular coil (162 ± 120 ng/mL) was comparable to that of eye drops (206 ± 116 ng/mL), and at day 4, the concentration was significantly higher (*p* = .001) in the eye drop group (299 ± 205 ng/mL versus 10 ± 11 ng/mL). After day 4, the concentration aqueous humor of the ocular coil group dropped below the detection limit whereas it could be measured in the eye drop group (52 ± 8 ng/mL and 94 ± 74 ng/mL for days 7 and 28, respectively).

The ketorolac concentration in plasma (Figure 2(c)) at 4 h was 10 times higher (*p* = .006) in the ocular coil group compared to the eye drop group (148 ± 128 ng/mL and 14 ± 9 ng/mL, respectively). At 24 h, the plasma concentration was equal for both groups (7 ± 5 ng/mL and 7 ± 3 ng/mL, for the ocular coil and the eye drop group, respectively). After day 4, the concentration in the ocular coil group dropped below the detection limit where the plasma concentration of the eye drop group was 16 ± 12 ng/mL, 16 ± 12 ng/mL, and 12 ± 9 ng/mL for days 4, 7, and 28, respectively.

### 3.2. Efficacy of the ocular coil compared to eye drops and no treatment

**Figure 3.**
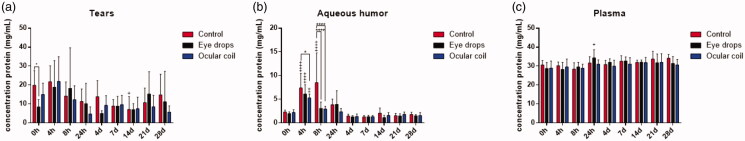
Total protein concentration in (a) tears, (b) aqueous humor, and (c) plasma. *N* = 8 rabbits per group, data are plotted as mean ± SD. ‘^+^’ Indicates significance compared to baseline condition, ‘*’ indicates a difference between two groups.

Efficacy was evaluated by measuring the total protein concentration and the concentration of cytokines in tears, aqueous humor, and plasma after inducing an ocular inflammation. The inflammation was treated using the ocular coil, eye drops, or left untreated. Figure 3 provides an overview of the total protein concentration in tears, aqueous humor, and plasma for the three animal groups. In tears (Figure 3(a)), no large differences in the total protein concentration were observed within the treatment groups. At baseline, however, difference between the control group and eye drops (*p* = .031) was seen, and at day 14, decrease of the total protein concentration was observed in the control group (*p* = .032).

In aqueous humor (Figure 3(b)), the total protein concentration strongly increased at 4 h from baseline in all animal groups. At 8 h, the total protein concentration was only elevated in the control group (*p* < .0001) and was back to baseline in the ocular coil group and the eye drop group. At 24 h, the total protein concentration was back at baseline level for all groups. Comparing the different groups, the total protein concentration in aqueous humor in the control group was higher compared to the ocular coil group at 4 h (*p* = .025), and higher compared to both treatment groups at 8 h (*p* < .0001).

In plasma (Figure 3(c)), a horizontal trend without peaks was observed. The total protein concentration is only higher when compared to baseline in the eye drop group at 24 h (*p* = .013).

**Figure 4.**
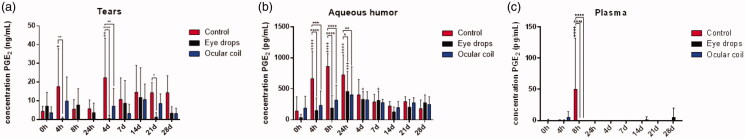
PGE_2_ concentration in (a) tears, (b) aqueous humor, and (c) plasma. *N* = 8 rabbits per group, data are plotted as mean ± SD. ‘^+^’ Indicates significance compared to baseline condition, ‘*’ indicates a difference between two groups.

The concentration of PGE_2_, an inflammatory mediator that is released immediately after inflammation, is depicted in Figure 4. In tears (Figure 4(a)), the concentration PGE_2_ at 4 h was higher in the control group compared to the eye drop group (*p* = .002). At day 4, the PGE_2_ concentration was higher in the control group compared to the eye drop group (*p* < .0001) and the ocular coil group (*p* = .007). At day 21, the PGE_2_ concentration was higher in the control group compared to the eye drop group (*p* = .048). In the control group, the PGE_2_ concentration was increased as compared to baseline at days 4 (*p* = .040) and 21 (*p* = .002).

In aqueous humor (Figure 4(b)), PGE_2_ concentrations increased significantly in the control group at 4 (*p* < .0001), 8 (*p* < .0001), and 24 (*p* < .0001) hours after induction of the inflammation. However, when treated with eye drops, a delayed increase of PGE_2_ was observed. Increase in PGE_2_ was observed at 24 h (*p* = .0005), at day 4 (*p* = .033), and at day 7 (*p* = .049) in the eye drop group, whereas treatment with the ocular coil did not result in significantly increased changes of PGE_2_. The control group had higher PGE_2_ levels compared to the eye drop group and the ocular coil group at 4 h (*p* < .0001 and *p* = .0002, respectively), 8 h (*p* < .0001 and *p* < .0001, respectively), and 24 h (*p* = .028 and *p* = .006, respectively).

In plasma (Figure 4(c)), the PGE_2_ concentration was undetectable in the majority of samples. The PGE_2_ concentration in the control group was increased at 8 h when compared to both treatment groups, as well as compared to baseline. No further changes compared to baseline or within the different groups were observed in plasma.

**Figure 5.**
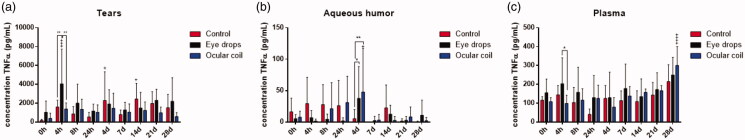
TNFα concentration in (a) tears, (b) aqueous humor, and (c) plasma. *N* = 8 rabbits per group, data are plotted as mean ± SD. ‘^+^’ Indicates significance compared to baseline condition, ‘*’ indicates a difference between two groups.

Figure 5 shows the concentration of TNFα, an inflammatory mediator related to the acute phase of inflammation, in tears, aqueous humor, and plasma. In tears (Figure 5(a)), the TNFα concentration at 4 h was higher in the eye drop group compared to the control group (*p* = .005) and the ocular coil group (*p* = .001). In tears, an increased TNFα concentration was observed in the control group at day 4 (*p* = .005) and at day 14 (*p* = .001) compared to baseline. In the eye drop group, an increase in the TNFα concentration was observed at 4 h (*p* = .005) compared to baseline.

In aqueous humor (Figure 5(b)), at day 4, the concentration of TNFα was higher in the eye drop group (*p* = .040) and the ocular coil group (*p* = .004) compared to the control group. The TNFα concentration as compared to baseline was also increased in the ocular coil group at day 4 (*p* = .017). In plasma (Figure 5(c)), the eye drop group shows increased TNFα at 4 h compared to the eye drop group (*p* = .028). Furthermore, the ocular coil has increased TNFα at day 28 (*p* < .0001) compared to baseline.

**Figure 6.**
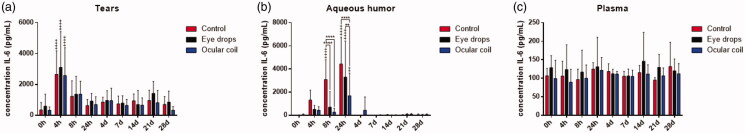
IL-6 concentration in (a) tears, (b) aqueous humor, and (c) plasma. N = 8 rabbits per group, data are plotted as mean ± SD. ‘^+^’ Indicates significance compared to baseline condition, ‘*’ indicates a difference between two groups.

The IL-6 concentration is plotted in Figure 6, IL-6 is also an important mediator for the acute phase of inflammation. In tears (Figure 6(a)), all three groups show elevated IL-6 concentrations at 4 h (*p* < .0001). However, no difference between the groups was observed for the different time points.

In aqueous humor (Figure 6(b)), the concentration of IL-6 is higher in the control group at 8 h compared to the eye drop group (*p* < .0001) and the ocular coil group (*p* < .0001), and is also higher at 24 h compared to the ocular coil group (*p* < .0001). At 24 h, the eye drop group also has a higher IL-6 concentration compared to the ocular coil group (*p* = .004). Compared to baseline, IL-6 is elevated in the control group at 8 h (*p* < .0001) and for all three groups at 24 h No changes in IL-6 levels have been observed in plasma (Figure 6(c)).

**Figure 7.**
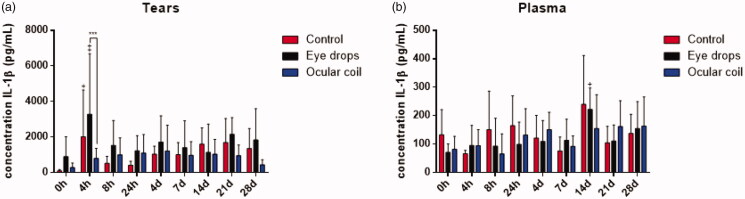
IL-1β concentration in (a) tears and (b) plasma. N = 8 rabbits per group, data are plotted as mean ± SD. ‘^+^’ Indicates significance compared to baseline condition, ‘*’ indicates a difference between two groups.

Figure 7 shows the IL-1β concentration in tears and plasma, IL-1β induces cyclooxygenase (COX) and is found to contribute to inflammatory pain. The concentration was below detection limit in aqueous humor. In tears (Figure 7(a)), the IL-1β concentration is higher in the eye drop group compared to the ocular coil group (*p* = .0005) at 4 h. Furthermore, increase in IL-1β is observed at 4 h in the control group (*p* = .021) and in the eye drop group (*p* = .002).

In plasma (Figure 7(b)), no differences between the groups were observed. However, the eye drop group shows an increased IL-1β concentration at day 14 (*p* = .011) compared to baseline.

## 4. Discussion

The effectiveness of commonly prescribed eye drop therapies is often compromised due to low patient compliance (Olthoff et al., 2009; Bertens et al., 2018). Therefore, we developed a noninvasive drug delivery device called the ocular coil (Bertens et al., 2018; Pijls et al., 2004, 2005, 2006, 2007). In this manuscript, we provided insights into the pharmacokinetics and efficacy of the ocular coil as an alternative to eye drops.

When comparing the pharmacokinetics of both delivery methods, higher ketorolac concentrations were found at 4 h in tears, aqueous humor, as well as plasma in the ocular coil group as compared to the eye drop group. Afterwards, ketorolac concentrations in both tears and aqueous humor from the ocular coil firmly decrease (approximately 100-fold), while ketorolac concentrations for eye drops remain similar. We believe that this difference is due to a difference in penetration into the anterior chamber (as concentration is a driver for penetration) and due to the lack of additives in the ocular coil to enhance penetration.

The ocular coil releases a single high dose (burst) of ketorolac where after drug release gradually lowers (Bertens et al., 2020). Applications that would greatly benefit from this burst release of drugs are acute inflammatory events such as (cataract) surgery induced inflammation or corneal ulcers that currently need fortified antibiotic application at an hourly dosing regimen during the first two days. Current drug release kinetics make the ocular coil not favorable for chronic diseases. Our results show that the ocular coil and eye drops achieve peak concentrations in aqueous humor of 2779.7 ± 1484.9 ng/mL and 983.4 ± 629.7 ng/mL, respectively, after 4 h. Bucci et al. reported peak concentrations of ketorolac in aqueous humor from cataract patients prior to surgery of 688.87 ± 749.6 ng/mL (Bucci & Waterbury, 2011). We would, however, expect higher concentrations in their study because they administer four additional eye drops one hour prior to surgery and because they sample quickly afterwards, while we sample 4 h later. Furthermore, since we need to stitch the ocular coil in the conjunctiva (and mock stitch the eye drop group), we expected that part of the administered ketorolac is used and thus less free ketorolac would be available.

In general, drug release via eye drops sharply peaks after each application and disappears quickly due to tearing and blinking (Urtti & Salminen, 1993; Lee et al., 2004; Hughes et al., 2005; Gaudana et al., 2010). In our experimental set-up, sampling always took place at the same time after eye drop application. Therefore, ketorolac levels were similar at different time points and the drug profile resembles a steady-state drug release instead of a peak pattern.

We tested the efficacy of the ocular coil after induction of inflammation by paracentesis. In the untreated control group we observed a three-fold increase in the total protein concentration in aqueous humor at 4 and 8 h after paracentesis. In particular a strong and steep increase in PGE_2_ concentration was observed. Already 4 hours after paracentesis, PGE_2_ concentrations were five-fold higher compared to baseline. The highest PGE_2_ concentrations were observed at 4, 8, and 24 h and slowly went back to baseline at day 28.

In the ocular coil and eye drops, PGE_2_ concentrations mildly increased (although not significantly different from baseline), whereas concentrations increased significantly in the control group. The largest treatment effects were observed at 4, 8, and 24 h after paracentesis. Interestingly, the effect was similar for the ocular coil as for eye drops. These results suggest that different drug release patterns (burst release followed by gradual drug release versus single peak drug dosing) can yield the same treatment effect.

Differences in PGE_2_ concentrations between untreated and treated groups were only observed during the first 24 h. After 4 days, PGE_2_ concentrations were back to baseline in all treated groups. This result raises questions regarding the intended treatment duration, which is currently set at 28 days for eye drops. Would a burst release of ketorolac be enough to halt the inflammatory cascade, or is prolonged exposure to the drug needed to achieve the optimal effect? This resembles a recent innovation in the pharmacological treatment of cataract surgery, where NSAIDS are provided during the surgery as an additive in the intraocular irrigation fluid. The use of a combination of ketorolac and phenylephrine (Omidria, Omeros Corp, Seattle, WA) was effective in the prevention of postoperative inflammation and in the reduction of cystoid macular edema following surgery (Visco & Bedi, 2020).

For the current study, we used a repeated sampling animal model. In this model, a trauma-induced acute ocular inflammatory response was provoked by drawing a large volume of aqueous humor (150–175 µL) (paracentesis) (Graff et al., 1998) followed by frequent sampling of small volumes (50 µL). The advantage of this model is that repetitive sampling within the same animal generates data at multiple (paired) time points. Thereby, limiting the total numbers of animals needed. A drawback of this model is that only limited volumes of tear fluid, aqueous humor, and plasma were available at each time point. Therefore, only few biomarkers could be tested thereby excluding the possibility to run technical replicates.

The performance of the drug-loaded ocular coil should be further validated in a clinical study. The *in vivo* pharmacokinetics of tears and in aqueous humor can be evaluated in patients undergoing regular cataract surgery (Walters et al., 2007; Bucci & Waterbury, 2009). This would clarify whether similar intraocular concentrations can be achieved as a comparison to ketorolac solutions added to the irrigation fluid during surgery (Omidria) and could be equally effective in preventing a postoperative inflammatory response.

## 5. Conclusion

In this study, we compared the pharmacokinetic profile and efficacy of the ocular coil with eye drops. The ocular coil showed a burst release during the first days where after drug release gradually lowered. Despite differences in their drug release pattern, we showed that both delivery methods are able to suppress an induced inflammation in a repetitive sampling model in New Zealand White rabbits. Applications of the ocular coil may be a promising alternative for eye drops in ocular diseases where a burst release can effectively prevent or treat ocular inflammation.
